# Cytoreductive Surgery and Hyperthermic Intraperitoneal Chemotherapy to Treat Pseudomyxoma Peritonei of Ovarian Origin: A Retrospective French RENAPE Group Study

**DOI:** 10.1245/s10434-023-14850-0

**Published:** 2024-02-10

**Authors:** Alexis Trecourt, Naoual Bakrin, Olivier Glehen, Witold Gertych, Laurent Villeneuve, Sylvie Isaac, Nazim Benzerdjeb, Juliette Fontaine, Catherine Genestie, Peggy Dartigues, Agnès Leroux, François Quenet, Frederic Marchal, Cecile Odin, Lakhdar Khellaf, Magali Svrcek, Sixte Thierry, Marilyn Augros, Alhadeedi Omar, Mojgan Devouassoux-Shisheboran, Vahan Kepenekian, the RENAPE Group, Julio Abba, Karine Abboud, Adeline Aimé, Koceila Amroun, Thierry André, Catherine Arvieux, Gerlinde Averous-Lang, Armelle Bardier, Houda Ben Rejeb, Jean-Marc Bereder, Philippe Bertheau, Frédéric Bibeau, Valérie Boige, Pierre-Emmanuel Bonnot, Olivier Bouché, Fatiha Bouhidel, Marie-Dominique Bouzard, Cécile Brigand, Chloé Broudin, Bertrand Celerier, Cécilia Ceribelli, Aurélie Charissoux, Anne Chevallier, Elise Clément, Julien Coget, Thomas Courvoisier-Clément, Marie Dazza, Cécile de Chaisemartin, Frédéric Di Fiore, Frédéric Dumont, Sylvaine Durand-Fontanier, Clarisse Eveno, Anne-Cécile Ezanno, Olivier Facy, Gwenaël Ferron, Johann Gagnière, Alexandre Galan, Maximiliano Gelli, Laurent Ghouti, Laurence Gladieff, Diane Goere, Jean-Marc Guilloit, Frédéric Guyon, Bruno Heyd, Marie-Françoise Heymann, Martin Hübner, Claire Illac-Vauquelin, Rachid Kaci, Amaniel Kefleysus, Vahan Kepenekian, Reza Kianmanesh, Marie-Hélène Laverrière, Valérie Lebrun-Ly, Jérémie H. Lefevre, Bernard Lelong, Anne-Isabelle Lemaistre, Brice Malgras, Pascale Mariani, Antoine Mariani, Pierre Meeus, Eliane Mery, Fabrice Narducci, Stéphanie Nougaret, David Orry, Pablo Ortega-Deballon, Brice Paquette, Julien Péron, Patrice Peyrat, Denis Pezet, Nicolas Pirro, Marc Pocard, Flora Poizat, Judith Raimbourg, Patrick Rat, Pauline Ries, Pascal Rousset, Pierre-Yves Sage, Hélène Senellart, Olivia Sgarbura, Cristina Smolenschi, Isabelle Sourrouille, Abdelkader Taibi, Williams Tessier, Emilie Thibaudeau, Yann Touchefeu, Bertrand Trilling, Jean-Jacques Tuech, Séverine Valmary-Degano, Sharmini Varatharajah, Véronique Verriele-Beurrier, Guillaume Vogin, Romuald Wernert, Benoit You

**Affiliations:** 1 Hospices Civils de Lyon, Hôpital Lyon Sud, Service de Pathologie, Lyon, France; 2 Université Claude Bernard Lyon 1, UR3738—Centre pour l’Innovation en Cancérologie de Lyon (CICLY), Lyon, France; 3 Hospices Civils de Lyon, Hôpital Lyon Sud, Service de Chirurgie Digestive, Lyon, France; 4 Hospices Civils de Lyon, Hôpital Lyon Sud, Service de Gynécologie, Lyon, France; 5 Institut Gustave Roussy, Service de Pathologie, Paris, France; 6 Institut de Cancérologie de Lorraine, Service de Biopathologie CHRU-ICL, Nancy, France; 7 Institut du Cancer de Montpellier, Service de Chirurgie Digestive Oncologique, Montpellier, France; 8 Institut de Cancérologie de Lorraine, Service de Chirurgie Oncologique, Vandoeuvre-lès-Nancy, France; 9 Institut du Cancer de Montpellier, Service de Pathologie, Montpellier, France; 10 Sorbonne Université, Assistance Publique—Hôpitaux de Paris, Hôpital Saint-Antoine, Service de d’Anatomie pathologique, Paris, France; 11 Center Hospitalier de Valence, Service de Pathologie, Valence, France; 12 Department of Surgery, Al-Amiri Hospital, Kuwait City, Kuwait

**Keywords:** HIPEC, Ovarian carcinoma, Cytoreduction, Ovarian pseudomyxoma, Mucinous tumor from teratoma, Peritoneal pseudomyxoma

## Abstract

**Background:**

Ovarian pseudomyxoma peritonei (OPMP) are rare, without well-defined therapeutic guidelines. We aimed to evaluate cytoreductive surgery (CRS) and hyperthermic intraperitoneal chemotherapy (HIPEC) to treat OPMP.

**Methods:**

Patients from the French National Network for Rare Peritoneal Tumors (RENAPE) database with proven OPMP treated by CRS/HIPEC and with histologically normal appendix and digestive endoscopy were retrospectively included. Clinical and follow-up data were collected. Histopathological and immunohistochemical features were reviewed.

**Results:**

Fifteen patients with a median age of 56 years were included. The median Peritoneal Cancer Index was 16. Following CRS, the completeness of cytoreduction (CC) score was CC-0 for 9/15 (60%) patients, CC-1 for 5/15 (33.3%) patients, and CC-2 for 1/15 (6.7%) patients. The median tumor size was 22.5 cm. After pathological review and immunohistochemical studies, tumors were classified as Group 1 (mucinous ovarian epithelial neoplasms) in 3/15 (20%) patients; Group 2 (mucinous neoplasm in ovarian teratoma) in 4/15 (26.7%) patients; Group 3 (mucinous neoplasm probably arising in ovarian teratoma) in 5/15 (33.3%) patients; and Group 4 (non-specific group) in 3/15 (20%) patients. Peritoneal lesions were OPMP pM1a/acellular, pM1b/grade 1 (hypocellular) and pM1b/grade 3 (signet-ring cells) in 13/15 (86.7%), 1/15 (6.7%) and 1/15 (6.7%) patients, respectively. Disease-free survival analysis showed a difference (*p* = 0.0463) between OPMP with teratoma/likely-teratoma origin (groups 2 and 3; 100% at 1, 5, and 10 years), and other groups (groups 1 and 4; 100%, 66.6%, and 50% at 1, 5, and 10 years, respectively).

**Conclusion:**

These results suggested that a primary therapeutic strategy using complete CRS/HIPEC for patients with OPMP led to favorable long-term outcomes.

**Supplementary Information:**

The online version contains supplementary material available at 10.1245/s10434-023-14850-0.

Pseudomyxoma peritonei (PMP) is a clinical entity defined by the intraperitoneal accumulation of mucinous ascites and mucinous tumor deposits originating from an intra-abdominal primary tumor. Almost all PMP are a consequence of an appendicular mucinous neoplasm; however, in 3% of PMP, the primary mucinous tumor is of ovarian origin.^1–6^ This remains difficult to prove, as ovaries are commonly invaded in appendiceal PMP. In ovarian PMP (OPMP), the tumor may be of germ cell origin (mucinous neoplasia arising from a mature teratomatous gastrointestinal-type epithelium) or of ovarian epithelial category (cystadenoma, borderline or malignant mucinous neoplasms).^7–10^

Accurate diagnosis of OPMP requires clinical, radiological and gastrointestinal endoscopic assessments as well as a histopathological examination of the appendix to exclude a primary digestive origin or an appendiceal mucinous neoplasia.^11^ A definitive diagnosis can only be performed after complete CRS, which includes the resection of the ovaries.^7^ Thus, the absence of digestive tract tumor, of primary appendiceal neoplasia, and the identification of a mature teratoma or Brenner tumor associated with the mucinous tumor in the ovary, favor an ovarian origin of the PMP. According to the National Comprehensive Cancer Network (NCCN) guidelines, the current preferred regimens to treat ovarian mucinous carcinoma is 5-fluorouracil/leucovorin/oxaliplatin, or capecitabine/oxaliplatin, or paclitaxel/carboplatin for stage IC, with possible addition of bevacizumab in stages II–IV.^12^ However, OPMP chemosensitivity to carboplatin and paclitaxel is controversial.^1–6, 12^ Although cytoreductive surgery (CRS) followed by hyperthermic intraperitoneal chemotherapy (HIPEC) is the standard treatment for PMP of appendiceal origin (APMP),^13–15^ there are no current guidelines for the treatment of OPMP. Reported cases of OPMP are single cases or small series, for which the treatment strategies are not always described.^8, 16–27^ Recently, Yan et al. reported a case-series of eight OPMP; six of them were treated using CRS± HIPEC and there were no recurrences in two cases, which suggested a potential therapeutic benefit of this procedure.^10^

The objective of this study was to evaluate the efficacy of CRS/HIPEC to treat OPMP patients through a retrospective analysis of the French National Network for Rare Peritoneal Tumors (RENAPE) database, based on a comprehensive pathological review. The second objective was to assess whether the origin of the ovarian tumor cell (i.e., teratomatous, non-teratomatous-mucinous epithelium, or unknown origin) could have an impact on the efficacy of this treatment.

## Methods

### Selection Criteria and Study Design

Clinically suspected OPMP cases were retrospectively selected, from 2000 to 2023, using medical records recorded in the French RENAPE database,^28^ labeled by the French National Cancer institute (INCa). According to the 2016 Peritoneal Surface Oncology Group International (PSOGI) classification consensus, OPMP were defined as an ovarian tumor with intraperitoneal accumulation of mucus including cellular or acellular mucinous ascites and peritoneal implants.^29^ All selected patients had normal colonoscopy and gastroscopy, an appendectomy, and underwent CRS/HIPEC. Moreover, when magnetic resonance imaging (MRI) and/or computed tomography (CT) scans were available at diagnosis, the absence/presence of pancreato-biliary primary tumor or urachal tumor was also noted.

Patients with a PMP of non-ovarian origin after pathological review (i.e., APMP pathologically proven) or for which the appendicular origin could not be excluded (i.e., appendix not assessable by histopathology at the time of the study) were excluded. This was crucial to avoid confusion between OPMP and APMP.^14^ Patients without proven histopathological peritoneal mucin deposit were also excluded, as it was impossible to grade the pseudomyxoma. Similarly, patients with non-mucinous primary ovarian tumors (e.g., endometrioid carcinoma) were also excluded.^7^

### Clinical Data Collection

Clinical and therapeutic data were collected. The Peritoneal Cancer Index (PCI) and the completeness of cytoreduction (CC) score were rated, as previously defined.^15, 30, 31^ The quality of cytoreduction was defined according to the CC score: CC-0: no macroscopic residual tumor; CC-1: residual tumor <2.5 mm; CC-2: residual tumor between 2.5 mm and 25 mm; and CC-3: residual tumor >25 mm.^31^ Cytoreductive surgery was considered complete when a CC-0 or CC-1 score was achieved. Intraperitoneal treatment consisted of HIPEC performed either with an open or closed abdomen technique, as previously described.^32^

The postoperative morbidity and mortality were evaluated according to the Clavien–Dindo classification during the 90 postoperative days and according to the National Cancer Institute (NCI) Common Terminology Criteria for Adverse Events (CTCAE) version 5.^32–34^ The intensive care unit (ICU) and total hospital length of stay were also recorded. Patients were followed-up every 3–6 months for 5 years, and then annually with clinical examination, serum blood tests and thoraco-abdomino-pelvic CT scan and/or peritoneal MRI completed with thoracic CT scan. Recurrence, defined as outcomes during follow-up, was diagnosed based on clinical, radiological, and/or histopathological findings, and was confirmed in multidisciplinary team meetings. The status at last news, total duration of follow-up, disease-free survival (DFS), and mean DFS were calculated.

All patients included in the French RENAPE database (NCT02834169) gave their informed consent. This study was conducted according to the Declaration of Helsinki.

### Pathological Examination

All samples from patients with supposed OPMP, including primary ovarian tumor, appendix, and peritoneal samples, were retrospectively reviewed by several gynecopathologists (AT, MDS, CG) and expert digestive pathologists (NB, SI, JF).^35^

The following gross criteria were collected for each tumor: laterality, size of the primary mucinous ovarian tumor(s), and the presence of a capsule rupture.

Since two accepted classifications of tumors responsible for PMP are available (digestive system tumors and female genital tract tumors),^7, 36^ primary tumors were classified according to both: the 5th digestive system tumor WHO classification.^36^ as low-grade (grade 1) and high-grade (grade 2) mucinous neoplasms (LGMN and HGMN, respectively) or signet-ring cell adenocarcinoma (grade 3), and the 5th female genital tract tumor WHO classification.^7^ as mucinous cystadenoma, mucinous borderline tumor (± intraepithelial carcinoma, ± microinvasion, ± microcarcinoma), or invasive mucinous adenocarcinoma (expansive or infiltrative invasion pattern). The presence of the following histopathological criteria were also noted on the primary ovarian tumor: mucinous lesion continuum (from benign cystadenoma to borderline or malignant mucinous neoplasm), mature teratoma or a Brenner tumor component, areas of pseudomyxoma ovarii and glandular rupture and ‘dirty’ tumor necrosis in mucinous glands.

Peritoneal metastases, regardless of the primary tumor, were graded according to the 5th edition of the WHO classification of digestive system tumors,^36^ i.e. acellular, grade 1 (hypocellular mucinous deposits, low-grade cytology, no infiltrative-type invasion), grade 2 (hypercellular mucinous deposits, high-grade cytological features, infiltrative-type invasion), or grade 3 (mucinous tumor deposits with signet-ring cells). The PMP were also categorized according to the pTNM Classification of Malignant Tumours (8th Edition).^37^: pM1a (peritoneal acellular mucinous deposits), pM1b (mucinous epithelial cells in peritoneal mucinous deposits), and pM1c (metastases in another site other than the peritoneum).

In all cases, the appendix was entirely included in paraffin blocks and histologically examined to exclude any appendiceal mucinous neoplasm.

### Immunohistochemistry

Immunohistochemical analyses were performed on the primary ovarian tumor when tissues were available (*n* = 12) using Ventana Benchmark automated immunostainer (Roche/Ventana, Oro Valley, AZ, USA), according to the manufacturer’s instructions, and with the following antibodies: anti-PAX8 (clone MRQ-50, ref. 760-4618, Roche/Ventana), anti-SATB2 (clone SATBA4B10, ref. MSK101-05, ZYTOMED, Berlin, Germany), anti-CK7 (clone SP 52, ref. 790-4462, Roche/Ventana), and anti-CK20 (clone SP 33, ref. 790-4431, Roche/Ventana).

Results were indicated as positive (+), negative (−), or focal/weak expression (+/−). For CK7 and CK20 expression, the intensity/extent of expression of both markers were compared with each other and indicated as CK7>CK20 (expression of CK7 higher than CK20), or CK20>CK7 (expression of CK20 higher than CK7), or CK7=CK20 (expression of CK7 equivalent to that of CK20). Ovarian mucinous epithelial-like (CK7>CK20, facultative PAX8+, and SATB2−), digestive-like (CK20>CK7, PAX8−, and facultative SATB2+) or no specific (CK7=CK20, PAX8 and SATB2 non-available) immunohistochemistry profiles were subsequently defined.

### Histopathological, Immunohistochemical and Clinical Correlation

Merging clinical, histopathological and immunohistochemical data, mucinous neoplasms responsive for OPMP were then classified into four distinct groups:

*Group 1:* Arising from an ovarian mucinous epithelial origin (no teratoma component, ovarian-like immunohistochemical profile, and/or mucinous neoplasm associated with a Brenner tumor component).

*Group 2:* Arising from an ovarian teratoma (teratoma component with digestive-like immunohistochemical profile when available).

*Group 3:* Likely arising from an ovarian teratoma, in which teratoma was not sampled during gross examination or was overgrowth of the mucinous tumor (no teratoma component, digestive-like immunohistochemical profile, clinical data and imaging supporting a primary ovarian origin).

*Group 4:* Non-specific group (immunohistochemical data not available at the time of the study, or no specific profile).

### Statistical Analysis

Descriptive statistics were used for the analysis of clinical and histopathological data, described as counts and percentages for dichotomous variables and medians and ranges for continuous values.

DFS was defined as the time from the first CRS/HIPEC procedure until recurrence or the last follow-up. Median time of follow-up and median DFS were calculated.

Survival analyses (DFS) were tested using the Kaplan–Meier method and log-rank test. The R statistical software (The R Foundation, Indianapolis, IN, USA; available from: http://www.r-project.org/) with the RcmdrPlugin.EZR package was used. A *p*-value <0.05 was considered statistically significant. The DFS calculation was performed to compare the survival between OPMP from teratoma/suspected teratoma origin (Groups 2 and 3) and ovarian/non-specific origin (Groups 1 and 4), in order to assess whether the ovarian tumor cell origin could have an impact on the efficacy of this treatment.

## Results

### Study Population

**Fig. 1:**
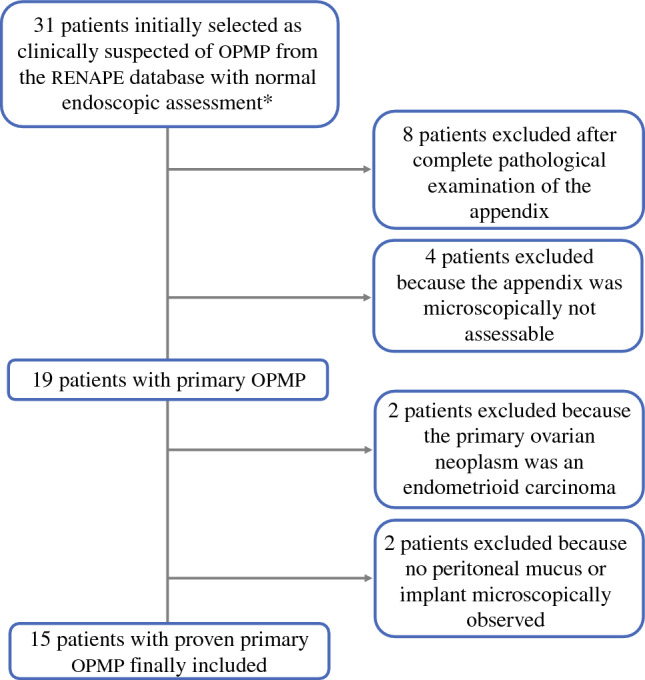
Case selection process. All patients included in the present study had been treated by CRS followed by HIPEC. In order to ensure with certainty the primary ovarian origin, all patients included had to have a normal digestive endoscopic and imaging assessment and a complete histopathological examination of the appendix (which had to be available for analysis at the time of the study). In addition, OPMP had to be histologically confirmed with the presence of mucus with or without tumor cells in the peritoneal samples. The primary ovarian tumor had to be mucinous (epithelial or developed on a teratoma). * Colonoscopy and gastroscopy. *CRS* cytoreductive surgery, *HIPEC* hyperthermic intraperitoneal chemotherapy, *OPMP* Pseudomyxoma peritonei from ovarian origin, *RENAPE* French National Reference Center for Rare Peritoneal Tumors

Between 2000 and 2023, 15 patients matching the inclusion criteria for OPMP were included (Fig. 1). The median age was 56 years (range 37.8–73.6) and the median preoperative CA125 dosage was 38 U/mL (range 22–800). At diagnosis, MRI and/or CT scans were available for 12/15 (80%) patients and showed no pancreato-biliary primitive or urachal tumor, while imaging performed during follow-up showed no pancreato-biliary primitive tumor. Two patients (13.3%) underwent a second surgical procedure (cases #13 and #14). At the time of the first CRS/HIPEC procedure, the American Society of Anesthesiologists (ASA) score was 1 in 3/14 (21.4%) patients, 2 in 8/14 (57.1%) patients, and 3 in 3/14 (21.4%) patients. The main characteristics of the patients and their treatment are fully reported in Table S1 of the electronic supplementary material (ESM) and summarized in Table 1.

**Table 1:** Clinical and pathological data

	All patients [*n* = 15]	Group 1^a^ [*n* = 3]	Group 2^a^ [*n* = 4]	Group 3^a^ [*n* = 5]	Group 4^a^ [*n* = 3]
*Clinical data*					
Median age at the time of CRS+HIPEC, years	56	57.3	54.9	57.2	56.3
Median PCI	16	16	26.5	14	7
CC score [*n* (%)]					
CC-0	60 (9/15)	2/3 (66.7)	3/4 (75)	4/5 (80)	0
CC-1	33.3 (5/15)	1/3 (33.3)	1/4 (25)	0	3/3 (100)
CC-2	6.7 (1/15)	0	0	1/5 (20)	0
HIPEC protocol [*N/n* (%)]					
Oxaliplatin alone	5/15 (33.3)	1/3 (33.3)	1/4 (25)	2/5 (40)	1/3 (33.3)
Mitomycin alone	5/15 (33.3)	0	3/4 (75)	2/5 (40)	0
Oxaliplatin + irinotecan	3/15 (20)	1/3 (33.3)	0	0	2/3 (66.7)
Mitomycin + cisplatin	2/15 (13.3)	1/3 (33.3)	0	1/5 (20)	0
Median hospitalization stay following CRS, days	20	25	29	13	25
Median hospitalization stay in ICU following CRS, days	5	7	10	1.5	7
Duration of follow-up, months	109	81.6	71.5	179.9	166.1
Median DFS, months	83.3	81.6	75.8	180.9	79.8
*Pathological data: primary ovarian tumor*					
Median tumor size, cm	22.5	23	20.5	15	30.5
Capsule rupture [*N/n* (%)]	7/11 (63.6)	2/2 (100)	2/2 (100)	3/5 (60)	0/2 (0)
Gland/cyst rupture in ovarian stroma [*N/n* (%)]	10/15 (66.7)	3/3 (100)	2/4 (50)	3/5 (60)	2/3 (66.7)
Continuum^b^ [*N/n* (%)]	10/15 (66.7)	3/3 (100)	1/4 (25)	4/5 (80)	2/3 (66.7)
Tumor necrosis in glands	0	0	0	0	0
5th WHO classification of digestive system tumors [*N/n* (%)]					
LGMN (grade 1)					
HGMN (grade 2)	12/15 (80)	1/3 (33.3)	3/4 (75)	5/5 (100)	3/3 (100)
	3/15 (20)	2/3 (66.7)	1/4 (25)	0	0
5th WHO classification of genital female tumors [*N/n* (%)]					
Mucinous cystadenoma	1/15 (6.7)	0	0	1/5 (20)	0
Borderline mucinous tumor	11/15 (73.3)	1/3 (33.3)	3/4 (75)	4/5 (80)	3/3 (100)
Invasive mucinous carcinoma (infiltrative pattern)	3/15 (20)	2/3 (66.7)	1/4 (25)	0	0
*Pathological data: peritoneal samples*					
PMP grading [*N/n* (%)]					
Acellular—pM1a	13/15 (86.7)	2/3 (66.7)	4/4 (100)	4/5 (80)	3/3 (100)
Grade 1—pM1b	1/15 (6.7)	0	0	1/5 (20)	0
Grade 3—pM1b	1/15 (6.7)	1/3 (33.3)	0	0	0

*CC* completeness of cytoreduction, *CRS* cytoreductive surgery, *DFS* disease-free survival, *HIPEC* hyperthermic intraperitoneal chemotherapy, *ICU* intensive care unit, *PCI* Peritoneal Cancer Index, *PMP* pseudomyxoma peritonei

^a^Group 1: mucinous neoplasms arising from an ovarian mucinous epithelial origin; Group 2: mucinous neoplasm arising from teratoma; Group 3: mucinous neoplasm arising from a probable ovarian teratoma; Group 4: non-specific tumor group

^b^Composed of benign ± borderline ± malignant mucinous carcinoma

### Gross Examination of Primary Ovarian Tumors

A total of 13/15 (86.7%) patients had a unilateral mucinous tumor, while 2/15 (13.3%) had bilateral mucinous tumors that were associated with at least a teratoma component. For patients with unilateral tumors, the neoplasm was located in the left ovary in 5/13 (38.5%) patients, the right ovary in 6/13 (46.1%) patients, and not specified in 2/13 (15.4%) patients.

The median size of ovarian mucinous tumors was 22.5 cm (range 10–35), with all tumors ≥10 cm in diameter. All data are presented in ESM Table S2 and Table 1.

### Histopathological Analysis

#### Primary Ovarian Tumors

A mucinous tumor arising from a mature teratoma was found in 4/15 (26.7%) patients, while no component of mature teratoma was observed in 11/15 (73.3%) patients. However, a contralateral mature teratoma (not associated with a mucinous neoplasm) was found in 1/15 (6.7%) patients. An ipsilateral Brenner tumor was associated with the mucinous neoplasm in 1/15 (6.7%) patients.

Classifications of ovarian tumors according to the 5th WHO classifications of both genital female tumors.^7^ and digestive system tumors,^36^ as well as histopathological analysis, are fully detailed in ESM Table S2 and Table 1. Regarding the WHO genital female tumor classification, no expansive pattern was observed.

#### Peritoneal Samples

PMP grading according to the 5th WHO classification of digestive system tumors.^36^ is presented in ESM Table S2 and Table 1. One patient (#5) had granulomatous peritonitis associated with PMP.

#### Histopathological Examination of the Appendix

No mucinous neoplasm of the appendix was observed after histopathological examination (data not shown).

### Immunohistochemical Analysis of Primary Ovarian Tumors

Ovarian tumors responsible for OPMP expressed PAX8 in 1/10 (10%) tumors, SATB2 in 3/10 (30%) tumors, CK7 in 6/12 (50%) tumors, and CK20 in 11/12 (91.7%) tumors. The comparison of CK7 and CK20 expression showed a CK7>CK20 expression in 3/12 (25%) tumors, a CK20>CK7 expression in 8/12 (66.7%) tumors, and a CK7=CK20 expression in 1/12 (8.3%) tumors.

Immunohistochemical data showed an ovarian mucinous epithelial-like profile in 3/15 (20%) tumors, a digestive-like profile in 8/15 (53.3%), and a non-specific profile in 1/15 (6.7%) tumors; immunohistochemical profiles were not available for 3/15 (20%) tumors. The results are presented in ESM Table S2 and Table 1.

### Histopathological, Immunohistochemical, and Clinical Correlation

**Fig. 2:**
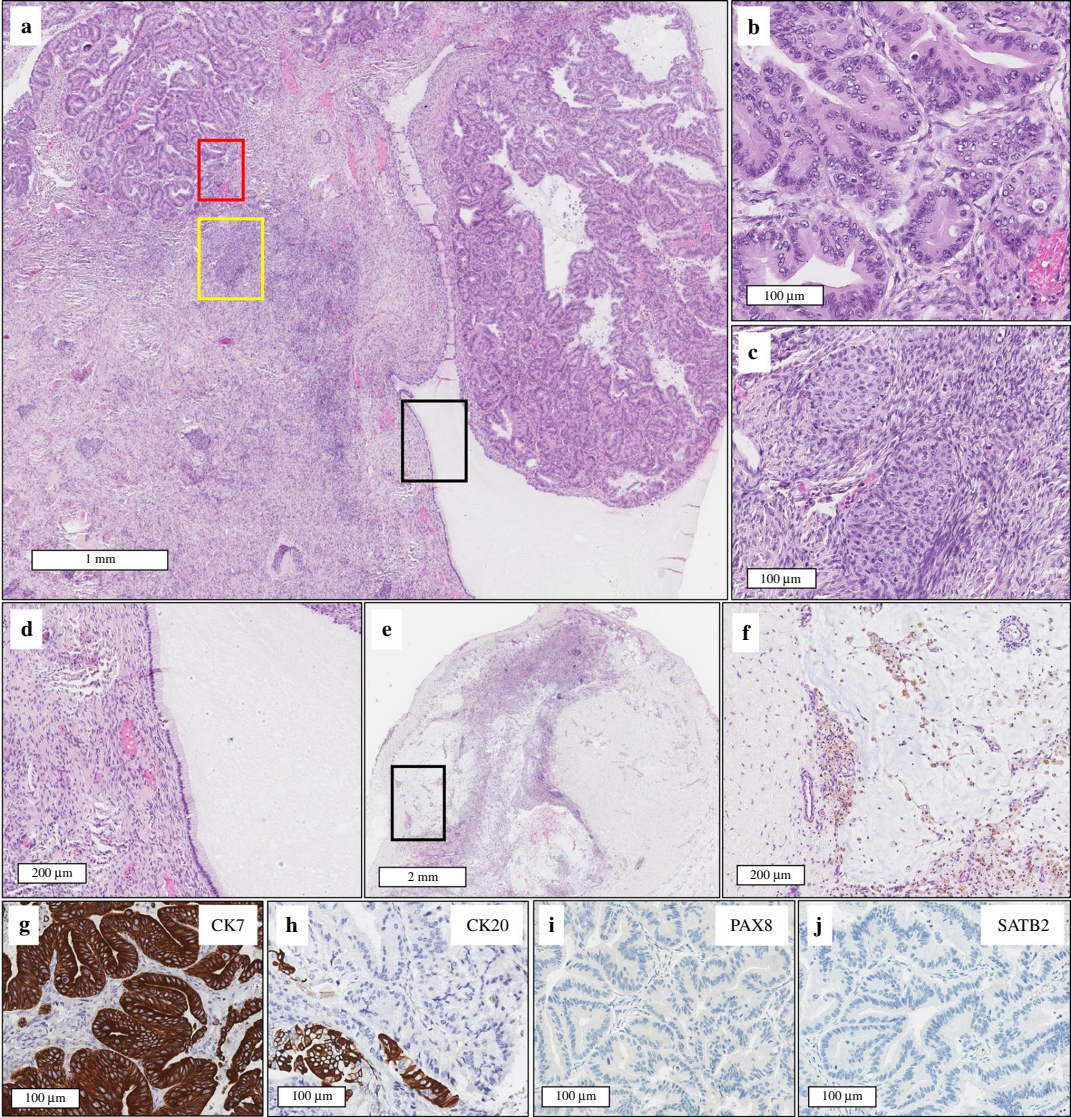
Pathological and immunohistochemical features of OPMP developed on ovarian mucinous epithelial neoplasm (Group 1; case #2). (**a**) HES (×20). Mucinous tumor with complex architecture at low magnification and several components: (1) a first component of infiltrative mucinous adenocarcinoma with small glands composed of atypical cells infiltrating the stroma (**b**) HES (×200] corresponding to the red frame of (**a**): (2) a contingent of benign Brenner tumor consisting of transitional nest within a fibromatous stroma. (**c**) HES (×200) corresponds to the yellow frame of (**a**); and (3) a component of benign mucinous cystadenoma consisting of unistratified mucinous cells without atypia. (**d**) HES (×100) corresponds to the black frame of (**a**). (**e**) HES (×10). (**f**) HES (×100). On peritoneum, the pseudomyxoma peritonei consists of mucus dissociating the peritoneal tissue, accompanied by numerous siderophages (**f** corresponds to the black frame of **e**). (**g**) CK7 (×200). **h** CK20 (×200). (**i**) (SATB2; ×200). (**j**) PAX8 (×200). the mucinous neoplasm expressed CK7 intensely and diffusely, with a weak/focal expression of CK20, but no expression of PAX8 and SATB2 (ovarian-like profile). *OPMP* Pseudomyxoma peritonei from ovarian origin, *HES* hematoxylin-eosin-saffron

**Fig. 3:**
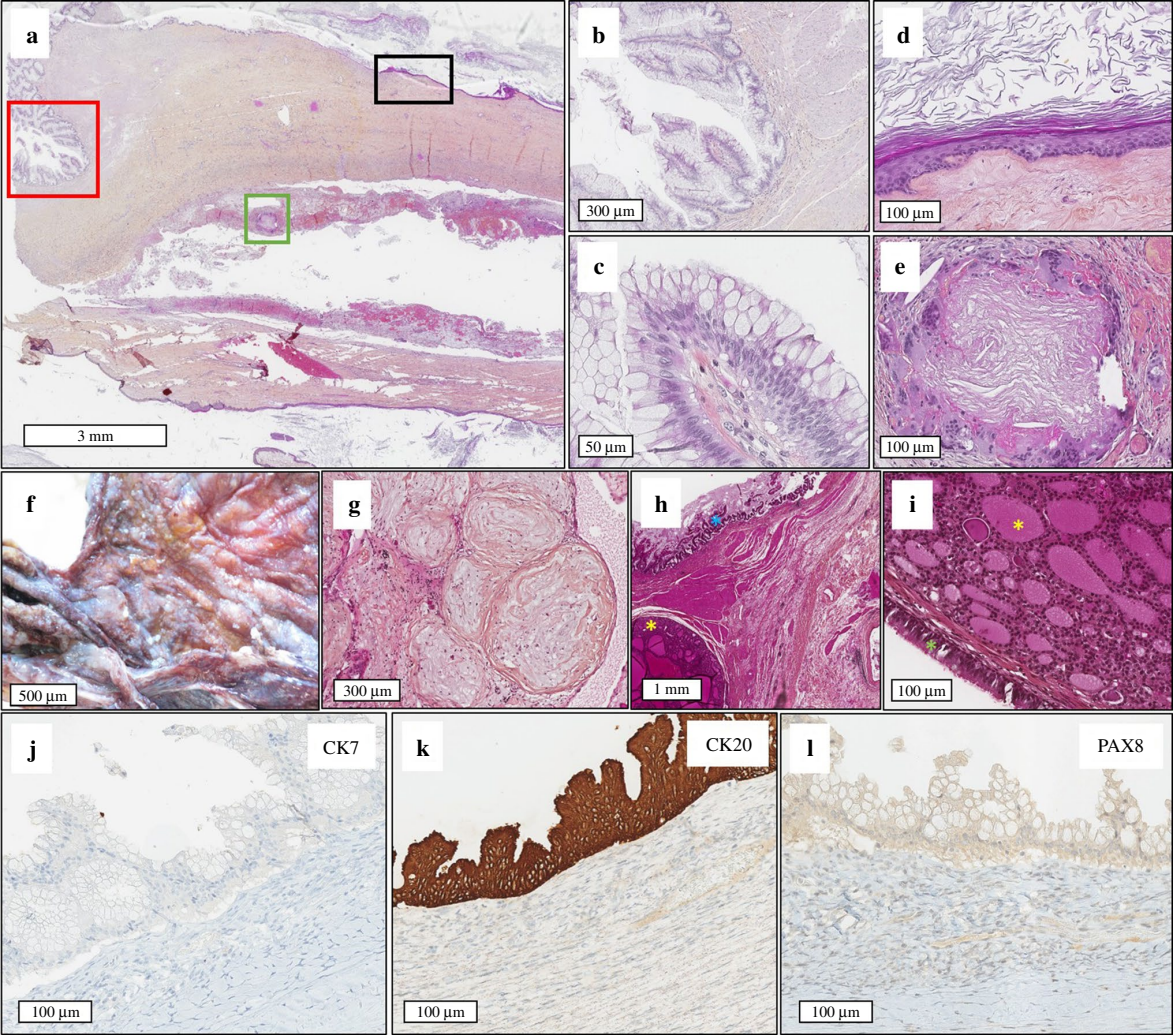
Pathological and immunohistochemical features of OPMP developed on ovarian teratoma (Group 2; cases #4 and #5). (**a**) HES (×10; case #4). Ovarian teratoma with several features: (1) a first component of low-grade mucinous neoplasm; red frame of (**a**) corresponds to (**b**) HES (×80) and (**c**) HES (×400), consisting of papillae lined with mucinous epithelium, without atypia or mitoses; (2) a second component consisting of well-differentiated squamous epithelium; black frame of (**a**) corresponds to (**d**) HES (×200), giving peritoneal granulomatosis. (**e**) HES (×200), consisting of keratin deposits surrounded by a granulomatous inflammatory reaction, whose differential diagnosis with carcinosis can be tricky. On peritoneum, pseudomyxoma peritonei gives a viscous gross appearance with granulations (**f**; case #3), with tissue dissociation by mucus. (**g**) HES (×80; case #3). Other teratomatous contingents were observed: thryoid tissue [yellow stars on (**h**) HES (×20; case #3) and (**i**) HES (×200; case #3)] adjacent to the mucinous neoplasm [blue star on (**h**)], or respiratory epithelium [green star on (**i**)]. (**j**) CK7 (×200). (**k**) CK20 (×200). (**l**) PAX8 (×200). The mucinous neoplasm expressed CK20 intensely and diffusely, with no expression of CK7 or PAX8 (digestive-like profile). *OPMP* Pseudomyxoma peritonei from ovarian origin, *HES* hematoxylin-eosin-saffron

**Fig. 4:**
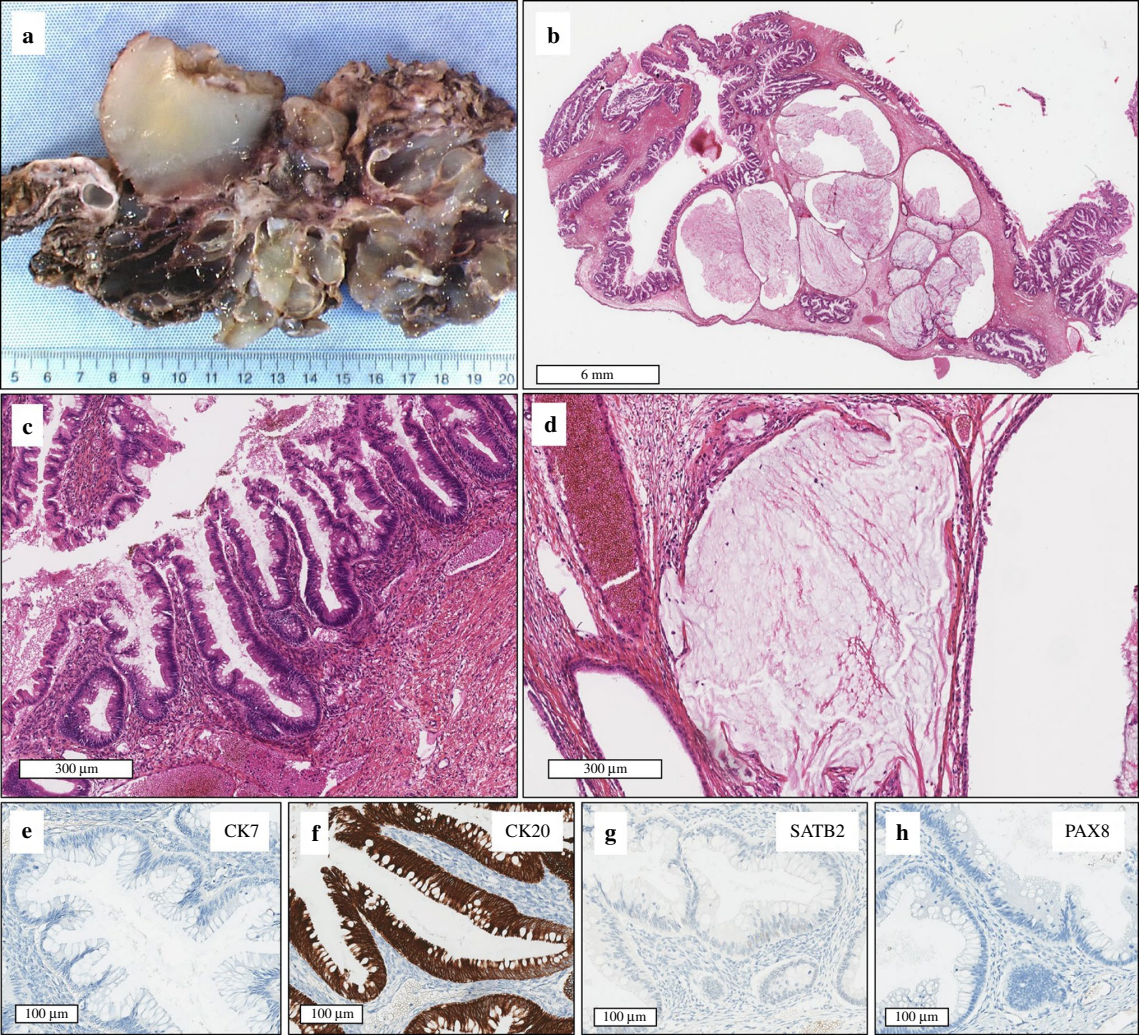
Pathological and immunohistochemical features of OPMP developed on ovarian mucinous neoplasms, likely corresponding to ovarian teratoma not sampled during gross examination (Group 3; case #9). (**a**) Gross examination of ovarian tumor after formalin fixation: the multi-cystic tumor, poorly limited with external vegetations and mucus within the cysts. (**b**) HES (×10). The tumor consists of numerous cysts and glandular ruptures in the ovarian stroma. (**c**) HES (×80). The tumor consists of papillae lined with mucinous epithelium. (**d**) HES (×80). Glandular ruptures in the ovarian stroma giving falsely infiltrative features. (**e**) CK7 (×200). (**f**) CK20 (×200). (**g**) SATB2 (×200). (**h**) PAX8 (×200). The CK20 expression was intense and diffuse in the mucinous neoplasm, with no expression of CK7 or PAX8. SATB2 expression was very focal and mild and was considered negative (digestive-like profile). *OPMP* Pseudomyxoma peritonei from ovarian origin, *HES* hematoxylin-eosin-saffron

Ovarian tumors responsible for OPMP were classified as Group 1 (mucinous neoplasms arising from an ovarian mucinous epithelial origin) in 3/15 (20%) tumors (Fig. 2); Group 2 (mucinous neoplasm arising from teratoma) in 4/15 (26.7%) patients (Fig. 3); and Group 3 (mucinous neoplasm arising from a probable ovarian teratoma not sampled during gross examination) in 5/15 (33.3%) patients (Fig. 4). A total of 3/15 (20%) tumors were classified as Group 4 (non-specific tumor group).

### Postoperative and Long-Term Outcomes

There were no postoperative deaths. Surgical complications occurred in 6/15 (40%) patients, among whom a severe outcome occurred in four patients; two of these patients underwent an additional surgery (one anastomotic leakage associated with hemorrhage, one abdominal wall abscess; grade 3b Clavien–Dindo, grade 3 CTCAE), one patient received a transfusion (anemia; grade 2 Clavien–Dindo, grade 2 CTCAE), and one patient had a pharmacological treatment (thromboembolic event; grade 2 Clavien–Dindo, grade 2 CTCAE). Two other patients presented hematological abnormalities during hospital stay: one patient presented with neutropenia and thrombocytopenia (grade 1 Clavien–Dindo, grade 1 CTCAE) and the other patient presented with febrile neutropenia and anemia (grade 1 Clavien–Dindo, grade 1 CTCAE).

The median length of hospital stay (conventional and ICU), median time of follow-up, and median DFS are presented in ESM Table S1 and Table 1.

**Fig. 5:**
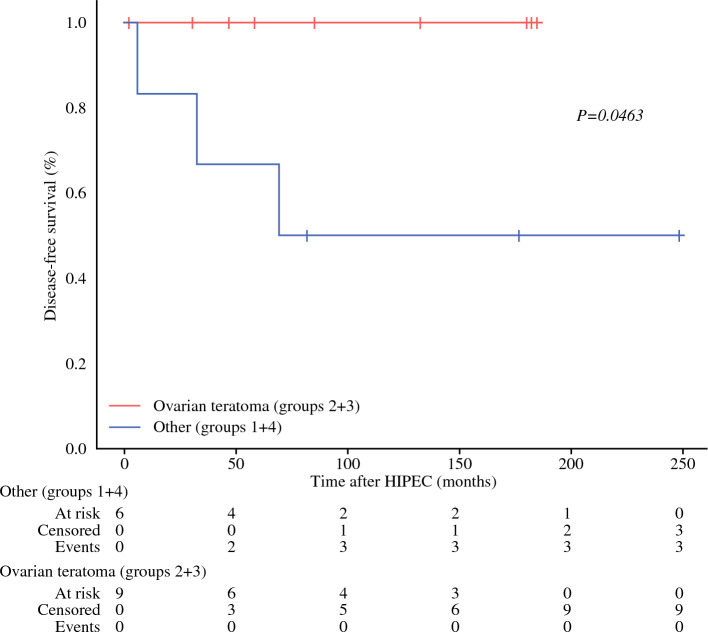
Survival analysis: comparison of the DFS of ovarian pseudomyxoma peritonei from teratomatous origin and other origins. The comparison of DFS between OPMP of teratoma/suspected teratoma origin (Groups 2 and 3) and ovarian/non-specific origin (Groups 1 and 4) showed a statistically significant difference (*p* = 0.0463). *HIPEC* hyperthermic intraperitoneal chemotherapy, *DFS* disease-free survival, *OPMP* Pseudomyxoma peritonei from ovarian origin

The comparison of DFS between OPMP of teratoma/suspected teratoma origin (Groups 2 and 3) and ovarian/non-specific origin (Groups 1 and 4) showed a statistically significant difference (*p* = 0.0463). The DFS for OPMP of teratoma/suspected teratoma origin was 100% at 1, 5, and 10 years, whereas the DFS for OPMP from ovarian/non-specific origin at 1, 5, and 10 years was 100%, 66.6%, and 50%, respectively. The results are presented in Fig. 5.

## Discussion

PMP of ovarian origin is an extremely rare and difficult entity to diagnose. A combination of clinical, endoscopic, gross, histopathological, and immunohistochemical findings are required to confirm the primary ovarian origin.^7–10^ The present study reported the largest series of patients with proven OPMP; all patients were successfully treated by CRS followed by HIPEC. A statistical difference was found between teratoma/suspected teratoma origin (groups 2 and 3) and ovarian/non-specific origin (groups 1 and 4) regarding the DFS.

To consider a PMP to be of ovarian origin, the cases were strictly selected; they had to present a normal digestive endoscopic assessment and a normal appendix after complete histopathological examination. A minority of the OPMP originated from an ovarian epithelial mucinous neoplasm with an ovarian-like expression profile (CK7>CK20; Group 1); one tumor was associated with a benign Brenner tumor. The majority were of probable teratomatous origin with a digestive-like expression profile (CK20>CK7; Groups 2 and 3), and a histologically visible mature teratoma was associated in four cases. In the remaining cases, the expression profile was not specific, and neither teratoma nor Brenner tumor was found. Thus, an extensive sampling and histopathological examination of the ovarian mucinous tumor is crucial, as the presence of a teratomatous component or a Brenner tumor would favor a PMP of ovarian origin.^38^ However, the mucinous malignancy can overgrow the initial mature teratoma; in one-third of cases, a digestive-like expression profile was found, without any teratomatous component. In the absence of primary digestive, appendicular, or pancreato-biliary tumor detected using endoscopy or imaging, as well as histologic appendicular examination, we speculated that these tumors might represent a mucinous neoplasia overgrowth in a mature teratoma. Moreover, the association of PMP with a granulomatous peritonitis around keratin debris (Fig. 3) could also be considered as an argument for a teratomatous origin. In old cases, whose material was no longer available, immunohistochemistry could not be performed, while an IHC panel is systematically performed today. This panel should incorporate anti-CK7, anti-CK20, anti-PAX8, and anti-SATB2 antibodies.^39^ However, in Group 2 (teratomas), SATB2 was not positive in all tumors and PAX8 expression was focal/weak in one case. Herein, the expression of PAX8 did not enable the tumor classification, likely due to the small size of our series, suggesting that the most important immunohistochemical feature is the comparison of the CK7/CK20 expression.

The criteria used for the classification of mucinous tumors are different in gynecological (ovarian mucinous tumors) and digestive pathology (appendicular mucinous tumors), which complexify the discussion between pathologists, gynecologists, and surgeons. Although it was suggested to classify and grade mucinous tumors arising from ovarian teratomas as appendicular mucinous tumors,^40^ it is not well defined as to how to classify and grade primary ovarian epithelial mucinous tumors responsible for OPMP (Group 1 herein). In the present study, tumors were classified according to the 5th edition of the WHO classification of both female genital tumors and digestive system tumors,^7, 36^ highlighting that LGMN were equivalent to mucinous cystadenomas and mucinous borderline tumors of the ovary, and that HGMN were equivalent to ovarian mucinous carcinomas (infiltrative pattern). Therefore, in the case of proven OPMP, it seems necessary to use both classifications, to allow all healthcare professionals involved in the complex management of these patients to understand each other.

However, no recommendations are currently available regarding the treatment of OPMP, as the disease is rare. In advanced ovarian cancer, platinum-based systemic chemotherapy is the standard regimen, regardless of the histological type. However, the mucinous type was reported to be associated with low chemosensitivity.^41–43^ and a concordant poor prognosis, worse than in patients with non-resectable high-grade serous carcinoma treated with only platinum-based chemotherapy (overall survival [OS] of 14.6 vs. 40.8 months; progression-free survival of 7.6 vs. 16.1 months),^44^ reinforcing the importance of surgical and locoregional treatments. In the present series, the efficacy of the treatment using CRS/HIPEC on patients with OPMP was reported. Moreover, a better DFS was found for OPMP of teratoma/likely-teratoma origin compared with ovarian origin/non-specific origin (*p* = 0.0463). The difference in DFS between these groups may be due to the type of epithelial proliferation. Mucinous adenocarcinomas arising from a teratoma are of colonic/appendiceal epithelium-type, while primary mucinous ovarian neoplasia are not supposed to arise from a colonic/appendiceal-type epithelium, as this does not exist in the ovary. CRS/HIPEC is the standard treatment for APMP.^13–15^ Thus, it is not surprising that a better response to this regimen is found for OPMP of teratomatous origin (colonic/appendiceal-type epithelium) compared with OPMP of ovarian epithelial-type origin. In the literature, survival data for OPMP are scarce. Lee et al. reported a 5-year OS of 87% in a retrospective cohort of 35 cases,^45^ most of whom were treated by CRS alone. However, appendicular status was not available as the materials used originated from ovarian lesions, not from the appendix. Yan et al. reported eight OPMP cases treated by CRS ± HIPEC, and only two patients were disease-free after 25 and 83 months of follow-up.^10^ Baratti et al. reported 19 cases of extra-appendicular PMP, among whom nine cases were of ovarian origin.^6^; all patients underwent CRS/HIPEC and the 10-year OS for APMP and non-appendiceal origin was 63.4% and 62.0 %, respectively. Rufián-Andujar et al. reported 117 cases of PMP managed by CRS/HIPEC, of whom seven cases were of ovarian origin; for the OPMP subgroup, the reported 5-year OS was 80% and 5-year DFS was 66.7%.^26^ Herein, HIPEC regimens were heterogenous, and the majority were oxaliplatin and mitomycin alone. However, due to the high rates of severe morbidity, HIPEC protocols were replaced by cisplatin-based regimens, with a concomitant reduced risk of renal failure thanks to sodium thiosulfate intravenous perfusion.^15, 46^ Recently, the PSOGI led an international evidence-based consensus to define the recommended HIPEC protocol in such clinical situations (submitted).

This study has limitations inherent to its retrospective design, such as the presence of missing data or materials. Another limitation is that the main patients reported herein presented OPMP with acellular peritoneal mucus deposits (pM1a), which likely contributed to an increased survival. Moreover, as OPMP is a rare disease, this case series was small in size and no control group could be identified within the French RENAPE group. Due to the limited data available on OPMP, mainly described as case reports, we were unable to find in the literature a series of OPMP treated by CRS alone to compare the results of the present series. One study reported two patients with OPMP treated with CRS alone (as patients could not receive HIPEC), followed by adjuvant chemotherapy and bevacizumab; one patient relapsed at 6 months,^21^ highlighting the importance of a locoregional approach. In other case reports, surgery-alone treatment did not lead to relapses, but the duration of follow-up was short,^16, 18, 19^ except for one patient followed-up 21 months after surgery.^23^ The addition of HIPEC could be especially useful in case of grade 3 PMP, which may relapse after surgical treatment alone.^24^ However, other studies are needed to confirm these hypotheses and to evaluate the oncological outcomes related to CRS/HIPEC, especially in multicenter studies, due to the rarity of this entity.

## Conclusion

A diagnosis of OPMP should be established following a rigorous methodology in order to exclude other primary origins, namely appendiceal and pancreato-biliary. Considering the poor survival of patients with advanced mucinous ovarian tumors treated by systemic chemotherapy alone, a locoregional approach combining CRS+HIPEC should be considered as it was associated with long-term median DFS in this series.

### Electronic supplementary material

Below is the link to the electronic supplementary material.

Supplementary file1 (DOCX 20 kb)
