# Supplementary material for: Cytoreductive Surgery and Hyperthermic Intraperitoneal Chemotherapy to Treat Pseudomyxoma Peritonei of Ovarian Origin: A Retrospective French RENAPE Group Study
Source: Ann Surg Oncol. 2024 Feb 10;31(5):3325–38. doi: 10.1245/s10434-023-14850-0 (PMC10997733; doi:10.1245/s10434-023-14850-0)
Supplement: Supplementary file 1 — Supplementary file1 (DOCX 20 kb) [file 10434_2023_14850_MOESM1_ESM.docx]

**Supplementary Table S1: Clinical data**

| **Patients #**  **(group number)** | **Age at the time of CRS+HIPEC (years)** | **ASA score** | **Pre-operative**  **CA 125 (U/ml)^a^** | **Preoperative chemotherapy** | **PCI** | **HIPEC  modality** | **CC score** | **Total hospital stay (days)** | **ICU stay (days)** | **Adverse event during**  **hospitalization** | **Status at last news, relapse (time to relapse in months)** | **Duration of follow-up (months)** | **DFS (months)** | **Median DFS (months)** |
| --- | --- | --- | --- | --- | --- | --- | --- | --- | --- | --- | --- | --- | --- | --- |
| **#1**^b^  **(group 1)** | 57.3 | 1 | 800 | no | 16 | oxaliplatin, closed, 42°C, 30 min | CC-0 | 38 | 9 | septic shock | NED | 81.6 | 81.6 | 81.6 |
| **#2**^b^  **(group 1)** | 73.6 | 1 | 27 | no | NA | mitomycin-cisplatin, closed, 42°C, 90 min | CC-0 | 10 | 5 | none | AWD^c^ (32.6m) | 32.6 | 32.6 |  |
| **#3**^b^  **(group 1)** | 54.1 | 2 | NA | no | NA | oxaliplatin-irinotecan, open, 43°C, 30 min | CC-1 | 25 | 7 | none | NED | 176.4 | 176.4 |  |
| **#4**^b^  **(group 2)** | 54.2 | 3 | NA | no | 26 | mitomycin, closed, 42°C, 60 min | CC-1 | 37 | 18 | thromboembolic | NED | 84.9 | 84.9 | 75.8 |
| **#5**^b^  **(group 2)** | 55.5 | 2 | 26 | no | 29 | mitomycin, closed, 42°C, 90 min | CC-0 | 21 | 2 | neutropenia, thrombocytopenia | NED | 58.1 | 58.1 |  |
| **#6**^b^  **(group 2)** | 37.8 | 1 | 42 | no | 0 | mitomycin, closed, 42°C, 90 min | CC-0 | 9 | 1 | none | NED | 46.7 | 46.7 |  |
| **#7**^b^  **(group 2)** | 59.7 | 2 | NA | yes (8 cycles of FOLFOX) | 27 | oxaliplatin, open, 43°C, 30 min | CC-0 | 60 | 31 | anastomotic leakage, haemorrhage | NED | 132.3 | 132.3 |  |
| **#8**^b^  **(group 3)** | 57.2 | 2 | 39 | no | 0 | mitomycin, closed, 42°C, 90 min | CC-0 | 11 | 2 | none | NED | 30.2 | 30.2 | 180.9 |
| **#9**  **(group 3)** | 48.4 | 2 | 37 | no | NA | mitomycin-cisplatin, closed, 42°C, 90 min | CC-2 | 13 | 1 | none | DOD (184.6m) | 184.6 | 184.6 |  |
| **#10**^b^  **(group 3)** | 38 | 3 | 57 | no | 14 | oxaliplatin, open, 43°C, 35 min | CC-0 | 19 | 7 | none | NED | 181.9 | 181.9 |  |
| **#11**  **(group 3)** | 71.7 | NA | NA | no | NA | oxaliplatin, open, 43°C, 30 min | CC-0 | 19 | NA | none | NED | 179.9 | 179.9 |  |
| **#12**^b^  **(group 3)** | 67 | 3 | 36 | no | 25 | mitomycin, open, 40°C, 90 min | CC-0 | 12 | 1 | none | Recent case | | |  |
| **#13**  **(group 4)** | 64.3 | 2 | NA | yes (2 cycles of FOLFOX) | NA | oxaliplatin, open, 42°C, 30 min | CC-1 | NA | NA | none | NED (one relapse after 69.2m, then no relapse since 96.9m) | 166.1 | 69.2 | 79.8 |
| **#14**^b^  **(group 4)** | 52.4 | 2 | 193 | yes (4 cycles of FOLFOX) | 7 | oxaliplatin-irinotecan, open, 42°C, 30 min | CC-1 | 24 | 3 | abdominal wall abscess | NED (one relapse after 6m, then no relapse since 79.8m) | 85.8 | 79.8 |  |
| **#15**^b^  **(group 4)** | 56.3 | 2 | 22 | no | NA | oxaliplatin-irinotecan, open, 43°C, 30 min | CC-1 | 26 | 11 | febrile neutropenia, anemia | NED | 248 | 248 |  |

Footnotes.

A total of 15 patients were finally included (seven patients from Centre Hospitalier Lyon Sud, Hospices Civils de Lyon, Lyon, France; four from Gustave Roussy, Paris, France; two from Institut du Cancer de Montpellier, Montpellier, France; one from Institut de Cancerologie de Lorraine, Nancy, France; and one from Hôpital Saint-Antoine, Assistance Publique des Hopitaux de Paris, Paris, France)

ASA score: preoperative American society of anesthesiology (ASA)-score; AWD: Alive with disease; CC score: Completeness of cytoreduction-score; DFS: Disease-free survival; DOD: Dead of disease; FOLFOX oxaliplatin, 5-fluorouracil and folinic acid; HIPEC: Hyperthermic intraperitoneal chemotherapy; PCI: Peritoneal cancer index; NA: Not available; NED: No evidence of disease.

^a^upper normal limit: <35 U/ml; ^b^Imaging (RMI/Scan) performed at diagnosis, showing no pancreato-biliary primitive tumor; ^c^Pulmonary relapse, undergoing chemotherapy at the time of the study.

**Supplementary Table S2: Pathological results.**

|  | **Gross findings** | | **Histopathology and immunohistochemistry** | | | | | | | |
| --- | --- | --- | --- | --- | --- | --- | --- | --- | --- | --- |
| **Patients # (group number)** | **Laterality and size of the tumor (cm)** | **Capsule rupture** | **Teratoma component** | **Glandular/ cyst rupture in ovarian stroma** | **Continuum^a^** | **Tumor necrosis in glands** | **PMP grading** | **IHC (mucinous tumor)** | **IHC profil** | **Histopathological final diagnosis**^b^ |
| **#1**  **(group 1)** | unilateral  NA: 18 | NA | no | yes | yes | no | acellular – pM1a | CK7+, CK20+ (CK7>CK20) | ovarian-like | borderline mucinous tumor / LGMN (grade 1) |
| **#2**  **(group 1)** | unilateral  RO: 23 | yes | no | yes | yes | no | acellular – pM1a | CK7+, CK20+ (CK7>CK20), PAX8-, SATB2- | ovarian-like | mucinous carcinoma (infiltrative pattern) + Brenner tumor / HGMN (grade 2) |
| **#3**  **(group 1)** | unilateral  RO: 30 | yes | no | yes | yes | no | grade 3 – pM1b | CK7+, CK20- (CK7>CK20), PAX8-, SATB2- | ovarian-like | mucinous carcinoma (infiltrative pattern) / HGMN (grade 2) |
| **#4**  **(group 2)** | unilateral  LO: 30 | NA | yes | no | no | no | acellular – pM1a | CK7-, CK20+ (CK20>CK7), PAX8-, SATB2- | digestive-like | borderline mucinous tumor (teratoma) / LGMN (grade 1) |
| **#5**  **(group 2)** | unilateral  LO: 10 | NA | yes | no | no | no | acellular – pM1a | NA | NA | borderline mucinous tumor (teratoma) / LGMN (grade 1) |
| **#6**  **(group 2)** | bilateral  LO: 22  RO: 11 | yes (LO) | yes (bilateral) | yes (bilateral) | no | no | acellular – pM1a | CK7-, CK20+ (CK20>CK7), PAX8-,  SATB2+ | digestive-like | bilateral borderline mucinous tumors (teratoma) / LGMN (grade 1) |
| **#7**  **(group 2)** | bilateral  LO: 19  RO: 26 | yes (bilateral) | yes, RO only | yes (bilateral) | yes | no | acellular – pM1a | LO: CK7+, CK20+ (CK20>CK7), PAX8+/-, SATB2- | digestive-like | LO: borderline mucinous tumor / LGMN (grade 1)  RO: mucinous carcinoma (infiltrative pattern; teratoma) / HGMN (grade 2) |
| **#8**  **(group 3)** | unilateral  RO: 15 | yes | no | yes | yes | no | acellular – pM1a | CK7-, CK20+ (CK20>CK7), PAX8-, SATB2- | digestive-like | borderline mucinous tumors / LGMN (grade 1) |
| **#9**  **(group 3)** | unilateral  LO: 25 | yes | no | yes | yes | no | acellular – pM1a | CK7-, CK20+ (CK20>CK7), PAX8-,SATB2- | digestive-like | borderline mucinous tumor / LGMN (grade 1) |
| **#10**  **(group 3)** | unilateral  LO: 23 | yes | no | yes | yes | no | acellular – pM1a | CK7-, CK20+ (CK20>CK7), PAX8-, SATB2- | digestive-like | borderline mucinous tumor / LGMN (grade 1) |
| **#11**  **(group 3)** | bilateral  LO: 15  RO: 1.5 | no | yes, RO only (without mucinous component) | no | no | no | acellular – pM1a | CK7+, CK20+ (CK20>CK7), PAX8-, SATB2+ | digestive-like | LO: mucinous cystadenoma / LGMN (grade 1)  RO: teratoma without mucinous neoplasm |
| **#12**  **(group 3)** | unilateral  RO: 13 | no | no | no | yes | no | grade 1 – pM1b | CK7-, CK20+ (CK20>CK7), PAX8-, SATB2+ | digestive-like | borderline mucinous tumor / LGMN (grade 1) |
| **#13**  **(group 4)** | unilateral  RO: 26 | no | no | yes | no | no | acellular – pM1a | NA | NA | borderline mucinous tumor / LGMN (grade 1) |
| **#14**  **(group 4)** | unilateral  RO: 35 | no | no | no | yes | no | acellular – pM1a | CK7+, CK20+ (CK20=CK7) | no specific profile | borderline mucinous tumor + intraepithelial carcinoma / LGMN (grade 1) |
| **#15**  **(group 4)** | unilateral  LO: NA | NA | no | yes | yes | no | acellular – pM1a | NA | NA | borderline mucinous tumor / LGMN (grade 1) |

Footnotes.

+: positive; -: negative; +/- : focal/weak expression. HGMN: High-grade mucinous neoplasm; IHC: Immunohistochemistry; LGMN: Low-grade mucinous neoplasm; LO: Left ovary; NA: Not available; PMP: pseudomyxoma peritonei; RO: Right ovary.

^a^Composed of benign +/- borderline +/- malignant mucinous lesion.

^b^According to both the 5^th^ WHO classification of genital female tumors and the 5^th^ WHO classification of digestive system tumors, respectively.
